# Comparison of high and low pulse energy dusting protocols using holmium: YAG laser in flexible ureteroscopy for treatment of renal stones

**DOI:** 10.1080/20905998.2024.2343181

**Published:** 2024-04-15

**Authors:** M. Elshazly, K.M. Zeinelabden, M. Aziz, H. Kandeel, M. Selim

**Affiliations:** a Faculty of Medicine Urology Department, Menoufia university, Shibin el Kom, Egypt; b Faculty of Medicine Urology Department, Kafrelsheikh university, Kafr el-Sheikh, Egypt

**Keywords:** Laser, dusting, high power, lithotripsy, holmium (Ho:YAG)

## Abstract

**Objectives:**

The management of renal stones, particularly those less than 2 cm in diameter, has changed with the development of endourological techniques, among which flexible ureteroscopy (FURS) using laser for lithotripsy has become a cornerstone. This study aims to compare the effectiveness of high pulse energy versus low pulse energy laser settings in renal stone dusting by Holmium YAG laser.

**Patients and Methods:**

This prospective randomized study was conducted between September 2021 and November 2023 to compare the efficacy and safety of high energy versus low-energy pulse settings using a Holmium: YAG laser dusting of renal stones less than 2 cm in diameter. A total of 174 adult patients were included, divided equally into high- and low-pulse energy groups, based on the energy settings of the laser high energy (ranged from 1.2–2.5 Joules and frequency of 8 hz) and low energy (less than 0.5 Joules and frequency ≥ 15 hz) using the dusting ‎technique with non-touch non-stop approach. The study sought to evaluate the impact of these settings on stone fragmentation efficiency, operative time, laser energy consumption, and postoperative outcomes, including stone-free rates and complications.

**Results:**

The study involved 174 patients who underwent renal stone lithotripsy and showed that using high pulse energy laser dusting settings significantly reduced operative times and more rapid dusting compared to low pulse energy settings, without affecting the stone-free rate. The study observed no significant differences in stone size or location between both groups. Minor postoperative complications were similar between both groups, indicating high pulse energy settings for lithotripsy dusting.

**Conclusion:**

The efficacy of high pulse energy dusting in enhancing stone removal during surgery, potentially reducing operative time. Further validation through larger-scale studies is needed to solidify these findings. This technique presents a promising solution, particularly in regions with limited resources where acquiring expensive laser equipment is challenging.

## Introduction

The global prevalence of urinary stone disease treatment has shown a steady rise [1] Within the armamentarium of urinary stone management, the laser has emerged as a cornerstone for lithotripsy in endourological procedures [2]. The trajectory of laser technology has witnessed a progressive evolution, transitioning from the established holmium: yttrium-aluminum-garnet (Ho: YAG) laser with pulse modulation to the advent of thulium fiber laser (TFL) and, more recently, the innovative pulsed thulium: YAG (p-Tm: YAG) laser [3]

Among the various treatment modalities available, laser lithotripsy has gained prominence due to its minimally invasive nature and high success rates in fragmenting stones. However, the debate persists regarding the optimal laser power settings for this procedure [4,5] While a specific consensus for laser settings in lithotripsy is not yet reached, contemporary approaches in laser lithotripsy focus on two primary strategies: fragmenting stones into smaller retrievable parts or breaking them into minute fragments, commonly referred to as ‘dust,’ facilitating the natural passage of smaller particles [4,5]. The choice between high power and low power settings in laser lithotripsy significantly influences treatment outcomes, including stone fragmentation efficiency, procedural time, and potential tissue damage. High-energy power laser settings offer rapid stone ablation capabilities, enabling quick fragmentation but potentially raising concerns about thermal injury to surrounding tissues. In contrast, low-energy power settings, while reducing the risk of tissue damage, might prolong the procedure and necessitate additional manoeuvres for complete stone clearance [6].

This study aims to compare high versus low pulse energy laser settings in renal stone lithotripsy using low power machines, Holmium YAG 30 Watts, examining their respective advantages, limitations, and overall efficacy.

By scrutinizing existing literature and recent studies, this study intends to provide a comprehensive understanding of the clinical implications of choosing optimal laser settings in urolithiasis.

## Methods

This was a prospective randomized study done from ‎September 2021 to November 2023. The study included 174 adult patients with ‎hard renal stones less than 2 cm with Hounsfield unit of ≥1000 who were ‎candidates for flexible ureteroscopy (FURS). Patients were randomly distributed ‎according to the setting of the power and energy of LASER into high and low ‎ pulse energy groups. Institutional Research Board approval was obtained. Written informed consent was obtained from all ‎patients. Patients less than 18 years were excluded, ureteral stones, stones with Hounsfield units less than 1000, stones in ‎the calyceal diverticulum and patients with coagulopathy were also excluded from the ‎study. ‎

### ‎ Preoperative assessment

‎ All patients had detailed medical history, examination, routine pre-operative ‎investigations including urine culture, pelviabdominal ultrasound, plain X ray ‎‎(KUB) and non-contrast CT scan.‎

‎ Preoperative urine culture was requested, and antibiotic prophylaxis was given (ceftriaxone 1 gm IV).‎

### Surgical technique

‎ After general anaesthesia, the patient was placed in the dorsal lithotomy position. ‎Regular cystoscopy was done with the placement of DJ ureteric stent preliminary on ‎the stone-affected side for 2–4 weeks before FURS. The second step was done 2 ‎to 4 weeks post-DJ insertion. After the removal of the DJ and insertion of the guide wire ‎diagnostic semi-rigid ureteroscopy was done to assess the ureter. Ureteric access ‎sheath was inserted (12 F/45 cm) (Coloplast, PORGES, France) on the wire under ‎fluoroscopy guidance. Retrograde access to the upper urinary tract using WiScope single-use flexible URS 7.4 fr, OTU Medical Inc, Union City, California, USA. Identification of the stone, 200 µm laser fiber lithotripsy was done. ‎

The LASER disintegration was done using Holmium YAG laser 30 Watts (Sphinx Jr.LISA laser – Katlenburg Lindau Deutschland two setting protocols high and low energy). The high energy ranged from (1.2–2.5 Joules and frequency of 8 hz) and low energy (less than 0.5 Joules and frequency ≥15 hz) dusting ‎technique with non-touch non-stop approach was done initially on intact stone. The lithotripsy stopped when small ‎stone fragments (<2 mm) were seen. Proper size double J stent (A 5-6Fr/26–28 cm) ‎was inserted routinely at the end of the procedure. All operative details as ‎operative time, fluoroscopy time, hospital stay, and intra- and postoperative ‎complications were recorded.‎

### Postoperative outcomes

‎ Postoperative complications measured by modified Clavien-Dindo classification [7]. Stone free rate (SFR) was assessed by plain abdominal X-ray (KUB) and ultrasound ‎after 1 week, and by spiral CT scan after 2 months, residual stone fragments (<3 mm) were considered insignificant. ‎

## Statistical analysis

### Statistical analysis of the data

Data was fed to the computer and analyzed using IBM SPSS software package ‎‎version 20.0. (Armonk, NY: IBM Corp). Categorical data were represented as ‎‎numbers and percentages. Chi-square test was applied to compare between two ‎groups, ‎alternatively. For continuous data, they were tested for normality by the ‎Kolmogorov–Smirnov Quantitative data were expressed as range (minimum and ‎maximum), mean, ‎standard deviation and median. Student t-test was used to ‎compare two groups for ‎normally distributed quantitative variables, meanwhile, on the ‎other hand, Mann Whitney ‎test was used to compare two groups for not normally ‎distributed quantitative variables. Significance of the obtained results was ‎judged at the 5% level.‎

## Results

One hundred and seventy-four patients with hard renal stones underwent flexible uretroscopy, and they were divided into two groups, high pulse energy (87 patients) and low pulse energy (87 patients) laser dusting groups. The high energy group included 67 males and 20 females, the low power group included 45 males and 42 females. There was no significant difference regarding to average ages between both groups; on the other hand, the high energy group showed significant higher BMI. Regarding stone size and stone Hounsfield units, there were no statistical significance between the two groups. Stone locations were variable between upper, middle, lower calyceal and pelvic with no significance among both groups (Table 1).

**Table 1.** ‎ Comparison between the two studied groups according to patient ‎demographics’ and stone characteristics.

	High (*n* = 87)	Low (*n* = 87)	p
**Gender**			
Male	67 (77.0%)	45 (51.7%)	<0.001*
Female	20 (23.0%)	42 (48.3%)	
**Age**			
Mean ± SD.	46.9 ± 12.7	45.7 ± 14.6	0.6
**BMI**			
Mean ± SD.	28.6 ± 4.0	25.9 ± 5.4	<0.001*
**Stone volume(mm**^**3**^)			
			0.9
Median (Min. – Max.)	1473 (390–5333)	1500 (400–4350)	
**Stone Hounsfield units**			
			0.3
Median (Min. – Max.)	1200 (1000–1700)	1180 (1000–1600)	
**Stone location**			
Upper calyx	23 (26.4%)	25 (28.7%)	0.9
Middle calyx	21 (24.1%)	21 (24.1%)	
Lower calyx	26 (29.9%)	25 (28.7%)	
Renal pelvis	17 (19.5%)	16 (18.4%)	

**Figure 1.**
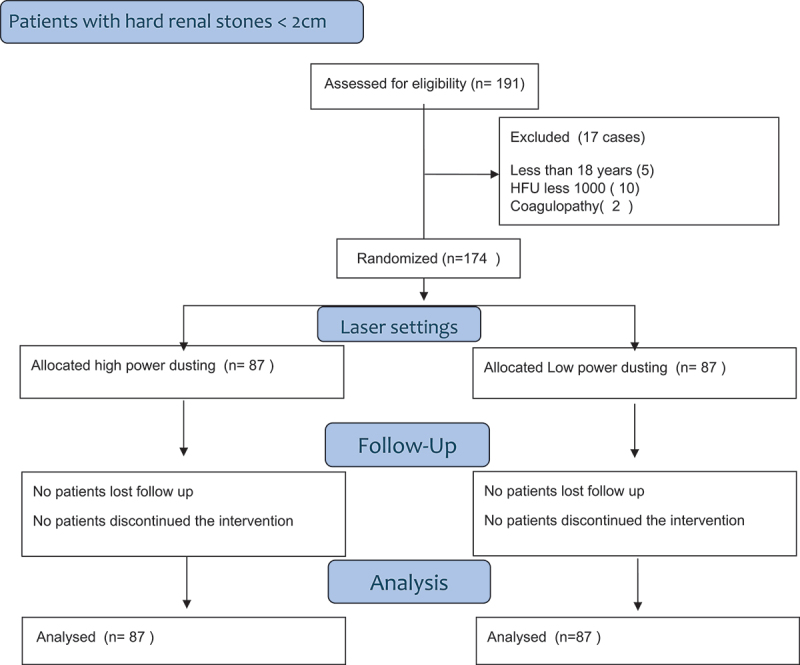
Patients CONSORT flow diagram.

Operative time was (47.9 ± 20.3 min) and (77 ± 32.5 min) for high and low energy group, respectively, that was statistically difference. Total laser energy and the laser consumption per/mm of stone were statistically higher in high energy than low energy group. The mean lasing duration was significantly shorter (12.5 ± 6.1 min) in high energy than (20.9 ± 8.8 min) in low power group. The high power showed significantly higher speed in stone fragmentation in comparison to low energy. The stone free rate was high in the high power and that was statistically significant. The severity of the complications was graded according to the modified Clavien-Dindo classification and there were no significant difference between both groups There was postoperative fever of low grade reported in five patients of high-energy group and seven patients of low power group which was not persistent and controlled by postoperative treatment also mild hematuria for few hours post procedure was reported patients improved spontaneously with no significance (Table 2). Patients with significant postoperative stone residual are followed up for spontaneous passage or second lock flexible URS after 2 months. Patient Data was summarized in patient consort flow diagram (Figure 1)

**Table 2.** Comparison between the two studied groups according to operative ‎data and post-operative outcome.

	High (*n* = 87)	Low (*n* = 87)	p
**Operative time (Min)**			
Mean ± SD.	47.9 ± 20.3	77 ± 32.5	<0.001*
Median (Min. – Max.)	50 [2] − 120)	75 [8] − 170)	
**Total LASER energy (Joules)**			
Mean ± SD.	20678.2 ± 13026.8	13713.2 ± 8758	<0.001*
Median (Min. – Max.)	17000 (4000–55000)	13500 (2000–35000)	
**LASER consumption/mm3 of stone**			
			<0.001*
Median (Min. – Max.)	23 [9] − 40)	10 [9–16]	
**Lasing duration (Min)**			
			<0.001*
Median (Min. – Max.)	12 [3] − 32)	19 [17] − 45)	
**Stone abilation speed**			
			<0.001*
Median (Min. – Max.)	11 [9–19]	6.7 (3.3–13)	
**Stone free rate (SFR)**			
No stone residual	15 (17.2%)	18 (20.6%)	0.6
Stone free	72 (82.7%)	70 (80.5%)	
**Complications**			
modified Clavien-Dindo classification			
Fever Grade II	5 (5.7%)	7(8%)	0.6 0.7
Hematuria grade I	3(3.4%)	2(2.3%)	

## Discussion

With recent advances in flexible ureteroscopy and laser technology, increasing experience, flexible ureteroscopy (FURS) has gained more popularity in treatment of renal stones [17].

The main target of lithotripsy is to efficiently disintegrate the stone with complete clearance. The techniques by which the laser disintegrates stones include fragmentation (high energy, low frequency) and dusting (low energy, high frequency) [10,11,18].

Fragmentation is done through a high energy and low frequency with fibre touching the stone, leading to fragmentation of stone into relatively larger particles followed by active stone fragment retrieval. This technique is usually used with hard stones like calcium oxalate monohydrate which is difficult to be dusted [12].

Dusting is done using low energy with high frequency to create tiny fragments with a high chance of spontaneous passage. Stone composition and hardness are important factors in the prediction of lithotripsy efficacy. Stone composition is not usually considered in pre-operative planning, and there is no consensus for definite laser setting in hard stones.

Dusting of hard stones with the known setting of dusting (low energy, high frequency) is usually difficult and time consuming. Our technique of changing the dusting setting into high energy, with the same dusting technique of no touch and continuous moving of laser fiber is a promising technique to efficiently dust hard stones in a shorter operative time [9,13,14].

Chawla and colleagues tested different laser settings in vitro on soda limestone using 1.0 J at a frequency of 20, 30, and 40 Hz, and an energy of 1.5 J and 20, 30, and 40 Hz. The settings of 1.0 J and 20 Hz were the most efficient [15]. Our study used a similar setting of 1.2–2.5 Joules for high pulse energy and less than 0.5 Joules for low pulse energy with non-contact laser lithotripsy but with the machine of 30 Watts power that can produce a maximum of 25 Hz; however, we used the setting proposed by Chawla initially on intact stone, not after stone fragmentation.

With high power new Holmium laser machines (more than 100 Watts) and Super pulsed Thulium laser machines dusting is more efficiently achieved. However, with low power Holmium laser machines, efficient dusting especially of hard stones is relatively challenging [16].

Shrestha utilized high power machine of 120 Hz reported the total energy used, and laser energy used to ablate 1mm3 of stone (Joules/mm3) was significantly higher in the HP group than in LP group (27.9 (16.4–46.2) J/mm3 vs 9.7 (5.3–17.7) J/mm3) (*p* < 0.01). Median (IQR) ablation speeds were 0.8 (0.5–1.3) mm3/s and 0.6 (0.4–1) mm3/s in the LP and HP groups, respectively. The median lasing time, operative time and stone free rate were similar in both the groups [6]. Our study showed comparable results with Total Laser energy, and the laser consumption of (23.1 ± 5.9 J/mm) VS (11.9 ± 2.5 J/mm) was statistically higher in the high energy than low energy group. The median ablation speed was significantly higher in high power in comparison to low energy. The mean lasing duration was significantly shorter (12.5 ± 6.1 min) in the high energy than (20.9 ± 8.8 min) in the low power group.

Our study showed a comparable SFR of 82.7% vs 80.5% for high power and low power, respectively. Previous studies comparing high power and low power reported similar SFR at both laser powers. Our operative time was significantly shorter in high power (47.9 ± 20.3 min) than low power (77 ± 32.5 min). Pietropaolo et al. reported operation durations of 52.02 ± 27.90 min in patients who used low power and 38.46 ± 22.88 min in patients using high power; also, our result showed no difference in complication between the two power groups in accordance with others [6,8,19]

Limitations of this study include need to study the thermal effect of higher energy and to include different types of stones as soft stones less than 1000 HU

## Conclusion

Our study proved that using this setting of high pulse energy dusting technique is a promising technique to achieve better dusting of hard stones in a shorter operative time. This needs to be validated in further larger studies. This is of practical significance, especially in countries with limited resources where expensive new high power Holmium laser, and new Thulium fibre laser machines are difficult to afford.
